# Onset and predictors of first-line antiretroviral therapy treatment failure among children in Ethiopia: a systematic review and meta-analysis

**DOI:** 10.1186/s12887-024-05324-7

**Published:** 2024-12-27

**Authors:** Molla Yigzaw Birhanu, Getamesay Molla Bekele, Bekalu Endalew, Simegn Alemu, Cheru Tesema Lashargie, Dereje Ayalew Birhanu, Assefa Mulualem, Selamawit Shita Jemberie

**Affiliations:** 1 Department of Public Health, College of Health Sciences, Debre Markos University, P.O.Box 269, Debre Markos, Ethiopia; 2 Department of Gynecology and Obstetrics, School of Medicine, Debre Markos University, Debre Markos, Ethiopia; 3 School of Public Health, Faculty of Health, University of Technology Sydney, Sydney, NSW Australia; 4 Department of Public Health, College of GAMBY Medical and Business, Addis Ababa, Ethiopia; 5 Department of Midwifery, College of Health Sciences, Debre Markos University, Debre Markos, Ethiopia

**Keywords:** First-line ART failure, Onset, Predictors, Children, Ethiopia

## Abstract

**Introduction:**

The emergence of First-line Antiretroviral Therapy (ART) regimens fails; it necessitates the use of more costly and less tolerable second-line medications. Therefore, it is crucial to identify and address factors that increase the likelihood of first-line ART regimen failure in children. Although numerous primary studies have examined the incidence of first-line ART failure among HIV-infected children in Ethiopia, national-level data on the onset and predictors remain inconsistent. Hence, this study was conducted to fill the gaps in determining the onset of first-line ART failure and its predictors among HIV-infected children in Ethiopia.

**Methods:**

Articles related to our topic of interest were searched using a systematic approach in national and international electronic databases. The retrospective follow-up cohort studies published in English up to 2022 were included. The data were extracted using a Microsoft Excel spread sheet and exported into Stata™ Version 17.0 for further management and analysis. The level of heterogeneity was quantified using I^2^ test together with a 95% confidence interval (CI). The incidence of the primary estimates was estimated using a random effects model in the Dersimonian-Lairedmethod. Subgroup analysis, Meta regression, and sensitivity analysis were computed to identify the source of heterogeneity but not explained. The predictors of first-line ART failure were explained using relative risk (RR) with 95% confidence interval (CI).

**Results:**

Ten studies having a total of 5446 children were included. The pooled onset of first-line ART failure was 3.18 (95% CI: 1.91, 4.44) per 100 child-years of observations. Those study participants who began ART at an advanced WHO clinical stage at ART initiation had a 3.05 (95% CI: 1.47, 6.36), having poor ART adherence had a 2.19 (95% CI: 1.29, 3.70), and having TB-HIV coinfection at ART initiation had a 1.43 (95% CI: 1.06, 1.94) times higher chance of experiencing first-line ART failure than their corresponding counterparts.

**Conclusion:**

The onset of first-line ART failure was high to achieve the 2030 UNAIDS target of ending the AIDS epidemic. Advanced WHO clinical stage, poor first-line ART adherence, and having TB-HIV coinfection were identified predictors. Hence, community HIV screening should continue to strengthen early ART initiation, and the attention of ART adherence should be kept to achieve ending the AIDS epidemic. The baseline tests and diagnosis, like TB diagnosis should be maintained for HIV-infected children while they begin ART.

**Supplementary Information:**

The online version contains supplementary material available at 10.1186/s12887-024-05324-7.

## Plain text

First-line ART failure is a significant global public health issue. In Ethiopia, there is no comprehensive data on the onset and predictors of first-line ART failure among school children on ART. This systematic review and meta-analysis aim to estimate the pooled onset and predictors of first-line ART failure among school children on ART in Ethiopia, using data extracted from 5,446 participants of 10 studies. Data analysis was computed using STATA version 17.0 statistical software. The presence or absence of heterogeneity was determined using a random effects model, and predictors were identified using a Meta-regression model. The study revealed that the onset of first-line ART failure in Ethiopia is high, posing challenges to achieving 2030 goals. Predictors of first-line ART failure included advanced WHO clinical stage at baseline, poor ART adherence, and TB-HIV coinfection at initiation.

## Introduction

HIV/AIDS leads to immune deterioration, making the body susceptible to secondary and opportunistic infections. To combat this, care and treatment such as Antiretroviral Therapy (ART) are essential. ART drugs are used to suppress the virus and restore immunity, thereby reducing the risk of HIV-related morbidity and mortality [1]. Without HIV care, including antiretroviral therapy, the progression of HIV infection in children is particularly swift [2]. ART drugs are lifelong medications that require continuous monitoring to maintain optimal viral suppression. While they are not a cure, they significantly reduce the risk of HIV-related morbidity and mortality [3]. The recommended ART regimens for children based on their weight are AZT or ABC + 3TC + EFV, AZT + 3TC + NVP, TDF + 3TC + EFV, TDF + 3TC + NVP, and ABC + 3TC + NVP. For 3-year-olds, the options include ABC or AZT + 3TC + LPV/r, ABC + 3TC + NVP, and AZT + 3TC + NVP [4] are the ART regimens given for children.

ART treatment failure is defined as the progression of the disease despite ART initiation, indicating an ineffective response to therapy. This failure limits future therapeutic options by reducing the available alternatives [5]. Therefore, it also affects the success of HIV treatment in meeting the UNAIDS goal of ending the HIV epidemic by 2030 [6]. This situation leads to the emergence and transmission of drug-resistant strains. Children who experience ART medication failure face increased side effects, including the spread of drug-resistant viral strains and higher mortality rates [7]. ART failure is an escalating problem that jeopardizes the efforts made for patients, communities, and the nation.

It is diagnosed using virologic, clinical, and immunological methods or as a combination of all three [8, 9]. Immune reconstitution inflammatory syndrome (IRIS) is a paradoxical condition where clinical deterioration happens even as the immune system improves, while treatment failure results in no immunologic progress [10]. Viral load testing is the gold standard for monitoring antiretroviral treatment failure. Immunological and WHO clinical criteria serve as secondary options for diagnosing ART treatment failure, as they have low sensitivity and positive predictive value in detecting viral failure [11]. Worldwide, it is estimated that of the 1.7 million children living with HIV, 65% received antiretroviral therapy (ART), and 57% achieved viral suppression by the end of 2021 [12]. Although numerous primary studies have examined the incidence and predictors of first-line ART failure, inconsistencies in the findings have made interventions challenging. This study aims to address incidence and predictors of first-line ART failure, which is important to prevent clinical events and subsequent morbidity/morality as well as providing valuable insights for policymakers, programmers, researchers, and healthcare practitioners.

**Research question**: what is the onset and predictors of first-line ART failure among Ethiopian children?

**Condition**: First-line ART failure

**Context**: Ethiopia

**Population**: EthiopianChildren on ART

## Methods

### Study area

Ethiopia is a Federal Democratic Republic with nine regional states (Afar, Amhara, Benishangul-Gumuz, Gambella, Harari, Oromia, Somali, Southern Nations Nationalities and People’s Region, and Tigray) and two city administrations (Addis Ababa and Dire Dawa). It has a total area of 1,100,000 km2 and is divided into zones, which are further subdivided into districts, which are further subdivided into kebeles, the lowest administrative divisions [13]. Ethiopia, with a population of approximately 112 million people, is Africa’s second most populous country (56,010, 000 females and 56, 069, 000 males in 2019) [14].

### Data source and searching strategy

This review was reported using the Strengthening the Reporting of Observational Studies in Epidemiology(STROBE) guideline [15], (additional file:1). We conducted a systematic search at electronic databases (PubMed/MEDLINE, CINAHL, EMBASE, Google Scholar, and Science Direct) from December 16, 2023to January 10, 2024. In addition to the databases, articles were also discovered by searching the reference lists of eligible studies. Two authors worked independently on the search (MYB and SSJ). Endnote X9 was used to retrieve and manage studies discovered after conducting a systematic search. ((First-line Antiretroviral Therapy [Text Word]) OR (Failure First-line Highly Active [Text Word])) OR (antiretroviral therapy resistance[Text Word])) OR (incidence[Text Word])) OR (predictors [Text Word])) OR (Antiretroviral Therapy [MeSH Terms])) AND (((((((HIV infected children[Text Word]) OR (HIV infected preschool[Text Word])) OR (Children living with HIV[Text Word])) OR (HIV positive children AND ((Ethiopia [Text Word]) OR (Ethiopia [MeSH Terms]))” were the searching engines used for searching articles.

### Eligibility criteria

#### Included criteria

All cohort studies conducted on incidence and predictors of first-line ART failure among Ethiopian children and Published in English were included.

#### Excluded

Articles which were not fully accessed after two email contacts with the corresponding authors and having similar title but different outcome variables were excluded.

**Design**: Systematic review and Meta-analysis

#### Screening procedure

Three authors (MYB, GMB, and SA) screened all titles and abstracts found in electronic databases independently. Disagreements were resolved through dialogue. Two authors (MYB and SSJ) screened the full text of the included articles independently, during which disagreements were resolved in the presence of a third author (GMB).

#### Quality assessment/risk of bias/

The quality of the included studies was evaluated using the Newcastle-Ottawa Quality Assessment Scale for cohort study (NOQAS) [16]. It depends on selection (Representativeness of the exposed cohort, Selection of the non-exposed cohort, Ascertainment of exposure and Demonstration that outcome of interest was not present at the start of study), Comparability (based on the design or analysis controlled for confounders), and outcome (Assessment of outcome, was follow-up long enough for outcomes to occur, Adequacy of follow-up of cohorts). The assessment tool guides that the articles that scored 3 or 4 stars in the selection domain AND 1 or 2 stars in comparability domain AND 2 or 3 stars in outcome/exposure domain were declared as “Good quality”, 2 stars in selection domain AND 1 or 2 stars in the comparability domain AND 2 or 3 stars in outcome/exposure domain were declared as “Fair quality”, and 0 or 1 star in selection domain OR 0 stars in comparability domain OR 0 or 1 stars in the outcome/exposure domain were declared as “poor quality” as a result, during the quality assessment of articles, all included articles were declared as having good quality.

#### Data extraction

The data were extracted using the data extraction checklist prepared from a Microsoft Excel spreadsheet. To ensure consistency and transparency, two authors (MYB and BE) extracted data independently using a predefined extraction checklist. The incidence of first-line ART failure among children, the study region, year of publication, sample size, follow-up period, and the first author name were extracted from the primary studies during data extraction.

#### Outcome variable and measures

The primary outcome of interest was the onset of first-line ART failure. It was calculated by considering the incidence of first-line ART failure and its standard error using a random effects model through the Dersmonian-laired method and quantified using I^2^ test. The second outcome of interest was predictors of first-line ART failure among Ethiopian children. It was again identified via log transformation of the primary studies; the effect size has been further computed. First, the RR of the primary studies was transformed into logRR to get the actual effect size, and its standard error was computed using lnlogRR. Hereafter, the binary Meta-regression model was fitted considering the logRR and lnlogRR to identify the predictors of first-line ART failure among Ethiopian schoolchildren. Finally, the strength of association between variables was presented using RR with a 95% CI.

#### Data management and analysis

For further analysis, the extracted data were exported to Stata™ Version 17.0 software. The pooled onset of first-line ART failure was estimated using a random effect model through the Dersmonian-laired method with I^2^ tests [17]. The standard errors (for the first and second objective) were computed from the reported estimates and population denominators using a normal distribution assumption. The presence of heterogeneity between studies was determined using the Cochran’s-Q test and quantified using I-square statistics; significant heterogeneity (I^2^ = 92.5% & *p*-value = 0.000) was found because heterogeneity is classified as high when its value is ≥ 50% [18]. To determine the presence or absence of publication bias, we used a funnel plot, which suggested the presence of publication bias due to the asymmetry of the scatterplot. We then used Egger’s linear regression test to confirm the presence of publication bias objectively, and it confirmed the absence of publication bias due to a *p*-value less than 0.05 [19]. An overall synthesized incidence of first-line ART failure was calculated and reported using a 95% confidence interval. To identify the source heterogeneity, subgroup analysis, sensitivity analysis, and meta-regression were computed but the heterogeneity was not explained. The study regions, publication year (before 2019 vs. after 2019), sample size (above mean vs. below mean), and follow-up period (≤ 6.7 vs. > 6.7 years) were the variables used to compute a subgroup analysis. Finally, the findings of systematic review and meta-analysis were presented in the form of texts, tables, and graphs like forest plots.

## Results

### Search results

**Fig. 1:**
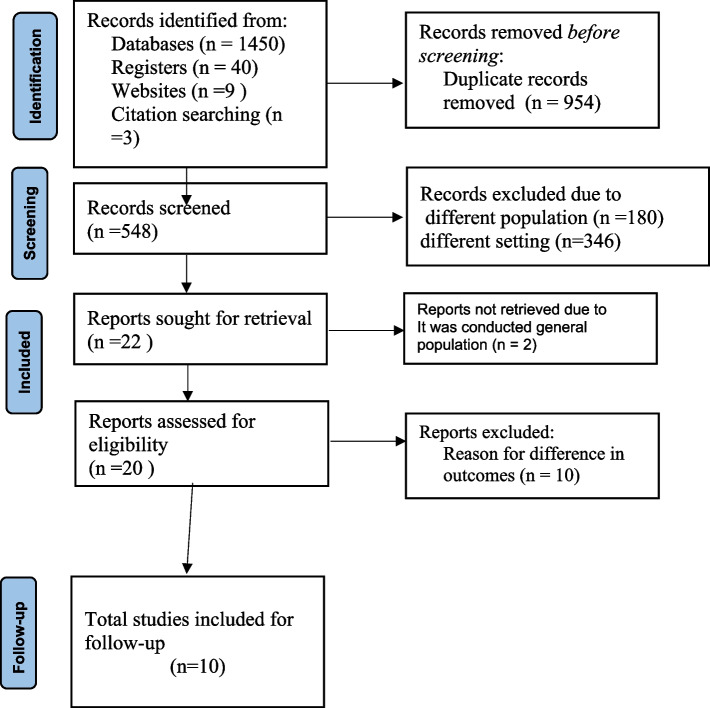
STROBE flow diagram of the included studies for first-line ART failure among Ethiopian children

 A total of 1502 studies were discovered through electronic database searches on PubMed/ MEDLINE, CINAHL, EMBASE, Google Scholar, and Science Direct, as well as organizational records and websites. Approximately 954 articles were excluded due to duplication, 346 articles were excluded due to differences in study setting/context [18–21], 180 articles were excluded due to differences in study population [22–24], 1 article was excluded was due to the study conducted on the general population [25], one article excluded due to having a different study design but it says predictors [23], 10 articles were excluded due to differences in outcome interest like incidence [26, 27]. Finally, 10 retrospective cohort studies were identified for inclusion and followed for the current systematic review and Meta-analysis (Fig. 1).

### Characteristics of the included studies

Ten studies involving a total of 5446 children were qualified for inclusion and further analysis in Ethiopia. These studies were conducted from three regions (Amhara region (*n* = 6) [28–33], Oromia region (*n* = 1) [29], Tigray (*n* = 1) [34]), and one city administration (Addis Ababa (*n* = 2) [10, 35]). All of the included studies were conducted through a retrospective follow-up method. The studies with the smallest and largest sample sizes, 250 and 1186, were conducted both in Addis Ababa, followed by a study conducted in Amhara region with a sample size of 710. The included studies’ follow-up periods ranged from 4 to 13 years, with 1298.32 to 13,379 child-years of ART failure free observations (Tables 1, 2, and 3).

**Table 1:** The characteristics of the included studies among Ethiopian children

Sn	Authors	Publication year	Region	cases	Sample size	PYO	Incidence	Follow-up period
1	Endale Zenebe et al. [29]	2021	Oromia	64	414	4145.75	1.54	4
2	Fassikaw Kebede et al. [33]	2021	Amhara	96	710	1935.24	0.41	4
3	Birkneh Tilahun Tadesse [32]	2021	Amhara	49	484	1299.73	4.97	7
4	Molla Azmeraw et al. [31]	2022	Amhara	315	459	1890	16.67	9
5	Migbar Sibhat et al. [34]	2020	Tigray	96	404	2765.38	3.47	4
6	Birtukan Aklog Yihun et al. [30]	2019	Amhara	49	402	1298.32	3.77	7
7	Malede Mequanent Sisay et al. [28]	2018	Amhara	63	824	4760.42	1.32	6
8	Endalk Birrie Wondifraw et al. [29]	2022	Amhara	47	336	27,058	2.1	13
9	Meseret Misasew [35]	2022	Addis Ababa	43	250	1780.54	2.42	7
10	Tigist Bacha et al. [10]	2012	Addis Ababa	167	1186	13,379	1.25	6

**Table 2:** The clinical characteristics of the study participants on ART

Characteristics	Categories	Frequency	Percentage
WHO clinical staging at baseline	Stage I/II Stage III/IV	239 217	52.41 47.59
CD4 count or CD4% at baseline	< 200cells/mm3 200- 500cells/mm3 ≥ 500cells/mm3	217 100 139	47.59 21.93 30.48
Hemoglobin level at baseline	< 10 g/L > 10 g/L	105 351	23.03 76.97
BMI	Normal Underweight	280 176	61.4 38.60
Height/age	Normal Stunting	308 148	67.54 32.46
Opportunistic infection	Yes No	89 367	39.04 60.96
Prophylaxis give	Not given Co-trimoxazole INH Both co-trimoxazole & INH	10 274 150 22	2.19 60.08 32.89 4.82
ART adherence	Good Fair Poor	125 89 242	27.41 19.52 53.07
Disclosure status	Disclosed Not disclosed	230 226	50.44 49.56
Drug side effects	Yes No	65 391	14.25 85.75
Does the regimen change	Yes No	157	34.43 65.57

**Table 3:** Multivariable cox proportional regression analysis model for first line ART failure

Variables	Survival status		Association (HR (95%CI)		*P*- value
	Event (105)	Censored (351)	CHR	AHR	
Residence					
Urban	34	308	1.00	1.00	**0.02**
Rural	71	43	2.21(1.56, 3.54)	5.30 ( 3.60 , 6.14)	
Disclosure status					
Yes	30	200	1.00	1.00	
No	75	151	1.56 (1.3, 5.89	1.61(0.47, 2.34	0.23
Baseline CD4 count					
< 200cells/mm3	66	181	1.00	1.00	
200- 500cells/mm3	28	72	0.34(0.23, 0.87)	0.27(0.85, 2.34)	0.98
≥ 500cells/mm3	11	128	0.23(0.12, 0.75)	2.12(0.95, 3.45)	0.79
WHO clinical staging					
Stage I/II	38	201	1.00	1.00	**0.01**
Stage III/IV	67	150	2.45(1.85, 3.12)	4.05 ( CI: 2.35, 5.24)	
height for age					
Normal	25	283	1.00	1.00	
Stunting	80	68	-1.2(-2.23, 0.45)	2.3 (0.68, 3.65	0.32
Adherence					
Good	32	93	1.00	1.00	
Poor	73	258	3.56(2.45, 6.31)	3.20 ( 2.21, 4.10)	**0.00**
Baseline OI					
Yes	71	18	1.00	1.00	
No	34	323	2.43(2.01, 3.15)	0.65(0.25, 1.89)	0.58

### The pooled onset of first-line ART failure

**Fig. 2:**
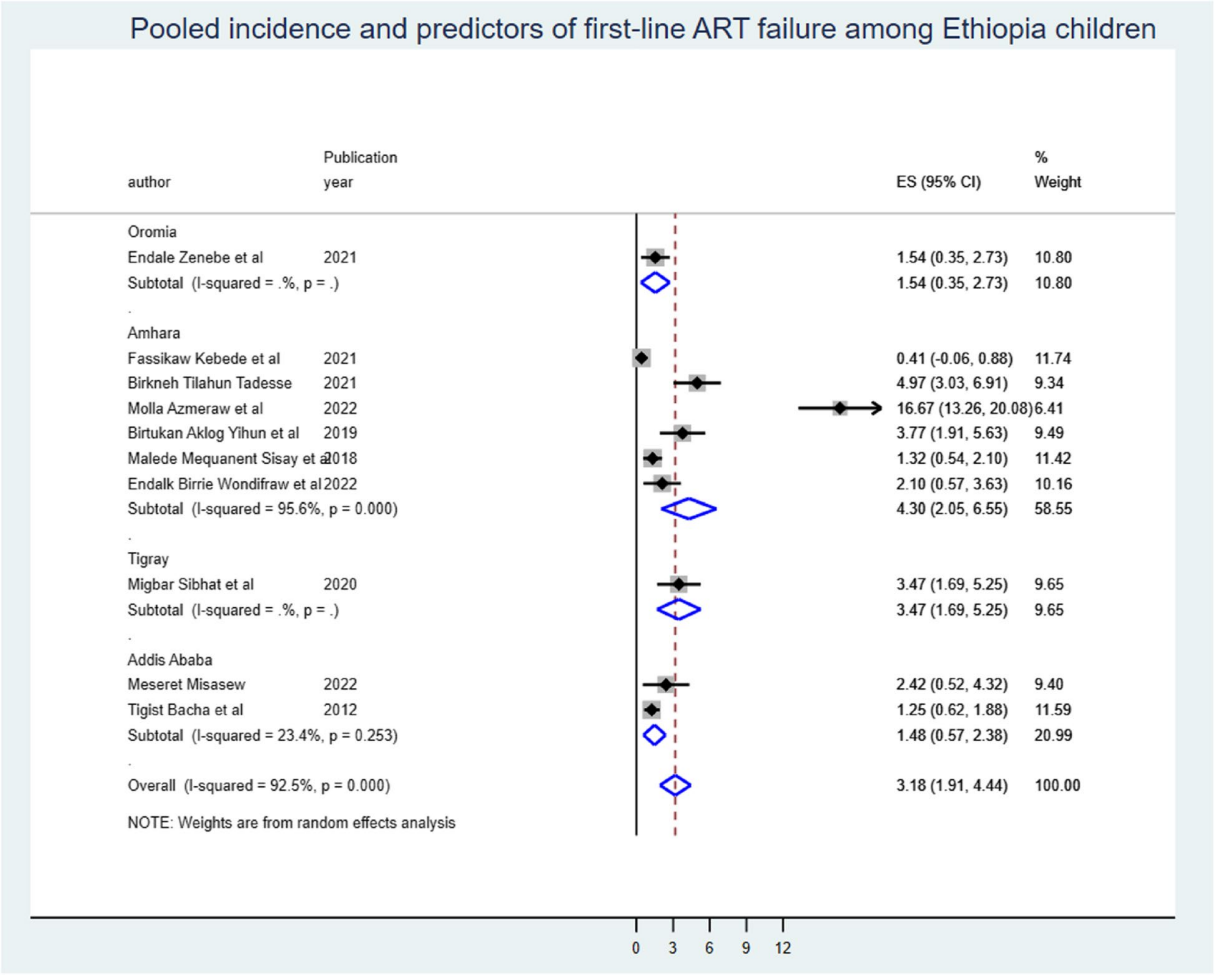
The overall pool onset of ART failure among Ethiopian children and by regional

The onset of first-line ART failure among Ethiopian children was 3.18 (95% CI: 1.91, 4.44) per 100 child-years first-line ART failure free observations. When we looked at it by region, Amhara had the highest (4.3 (95% CI: 2.05, 6.55) ART failure onset among children, followed by Tigray, 3.47 (95% CI: 1.69, 5.25), and Addis Ababa city administration had the lowest, 1.48 (95% CI: 0.57, 2.38) per 100 child-years of first-line ART failure-free observations (Fig. 2).

### Subgroup meta-analysis

**Fig. 3:**
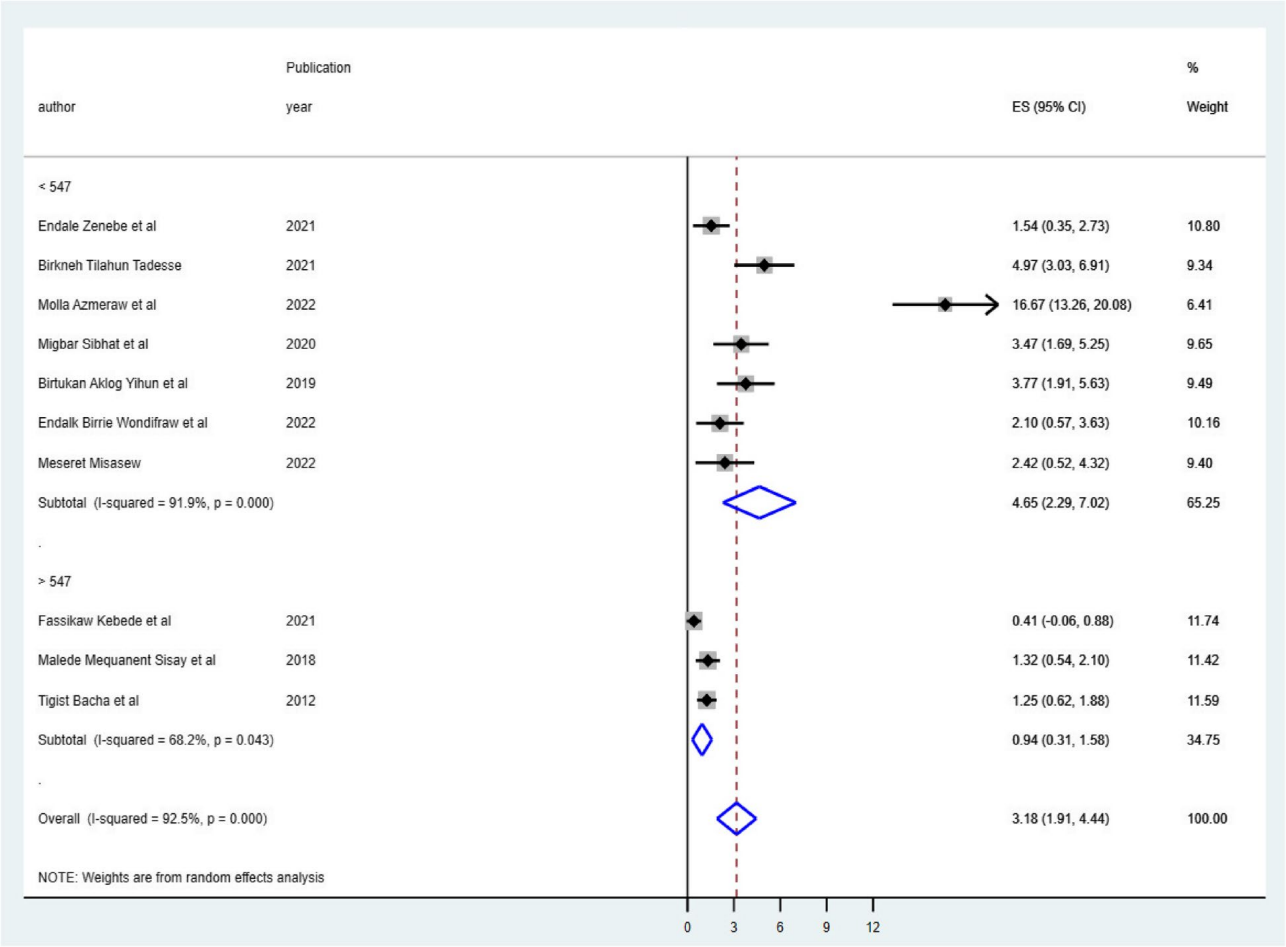
The subgroup analysis of ART failure among Ethiopian children using sample mean

**Fig. 4:**
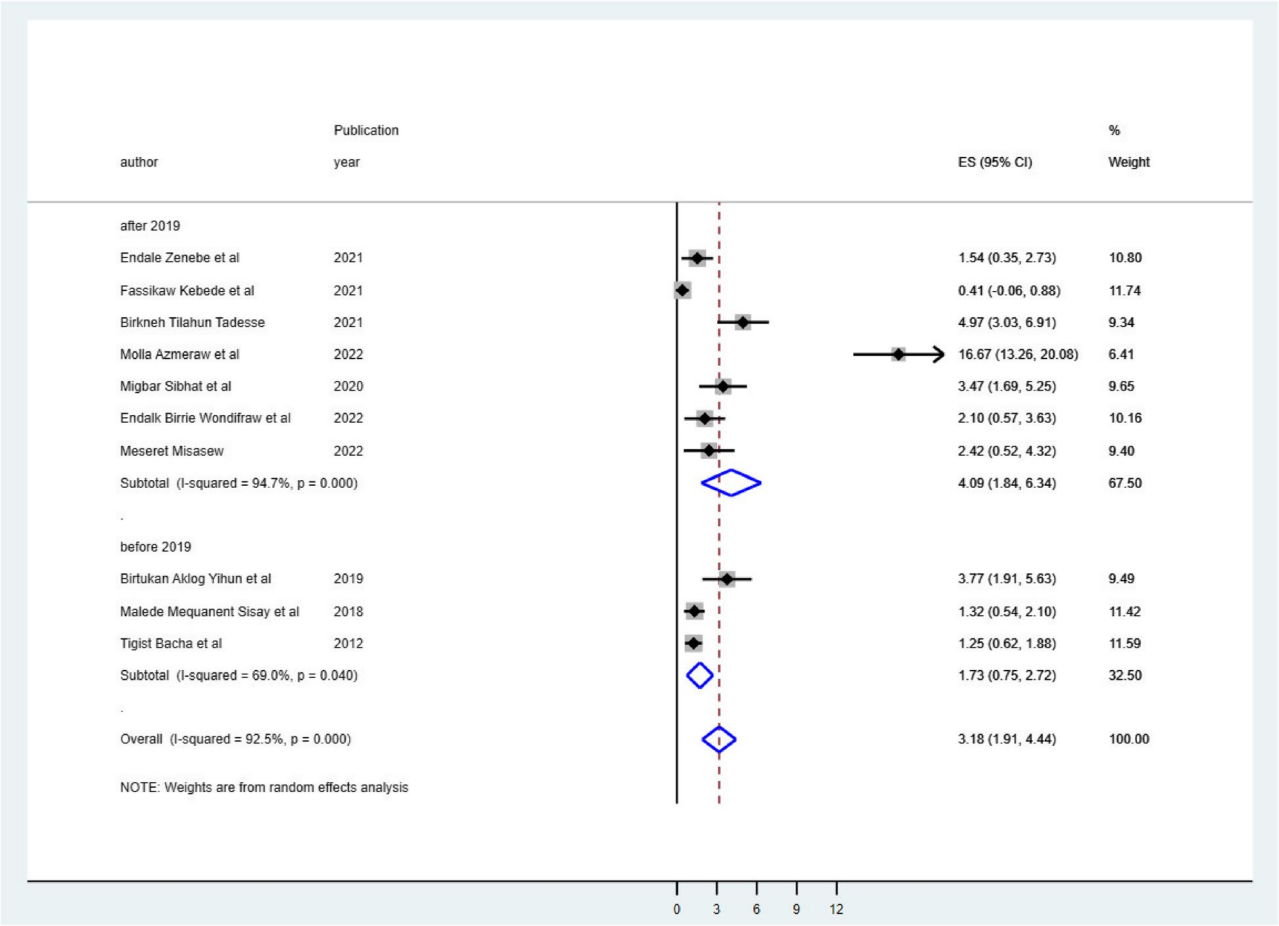
The subgroup analysis of ART failure among Ethiopian children using publication year

**Fig. 5:**
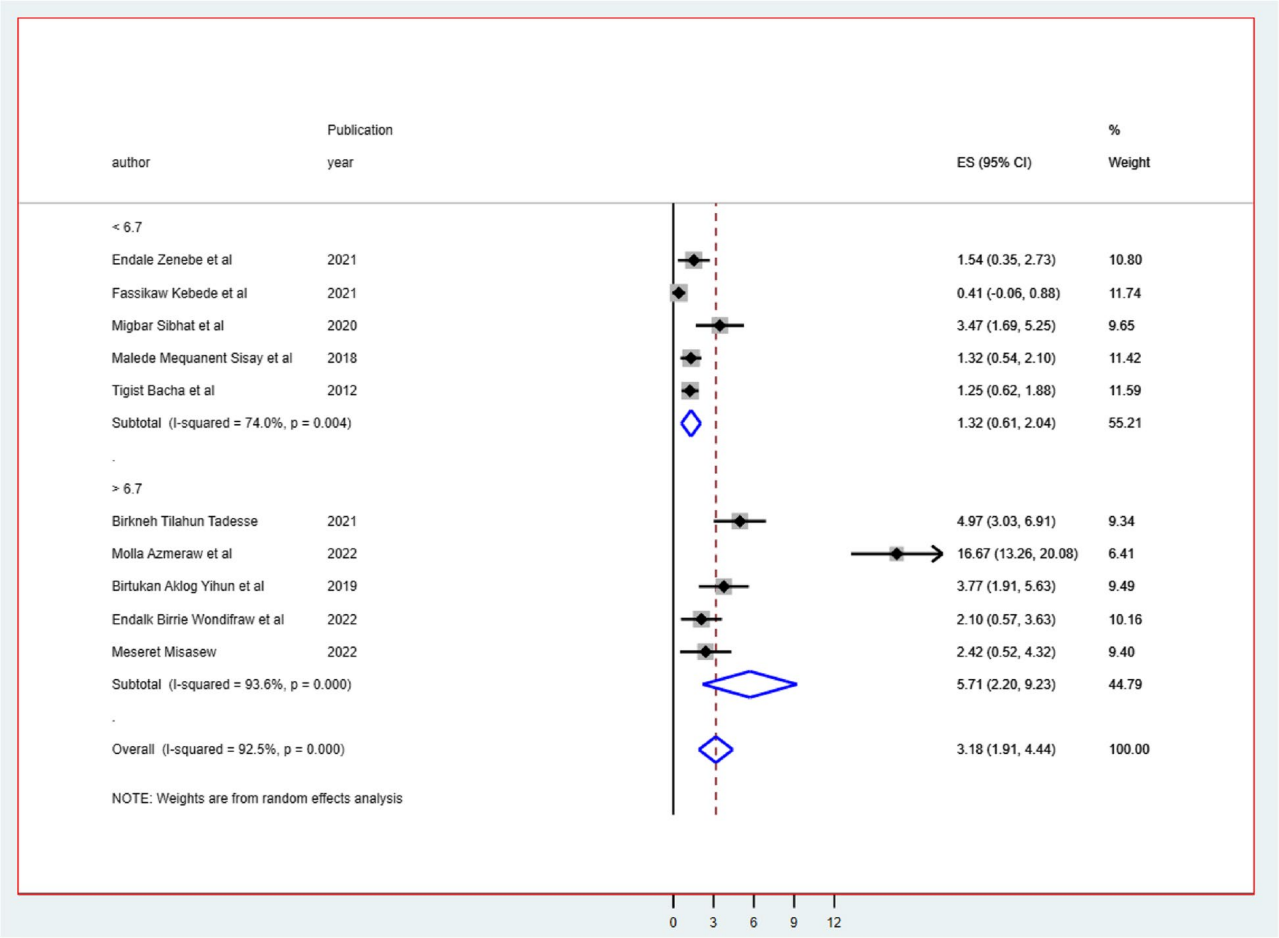
The subgroup analysis of ART failure among Ethiopian children using mean follow-up

Despite the presence of strong evidence supporting the existence of heterogeneity (using sample size for those whose sample size was less than the mean (< 547) I^2^ = 91.9 & > 547 I^2^ = 68.2 with *p*-value 0.0001 (Fig. 3), publication year (those published before 2019 I^2^ = 68.0 with *p*-value 0.04 & after 2019 I^2^ = 94.7 with *p*-value less than 0.001) (Fig. 4), and length of follow-up period ( those conducted less than the mean follow-up years (< 6.7 years) I^2^ = 74.0 with *P*-value 0.004, & those conducted greater than the mean follow-up years (> 6.7 years) I^2^ = 93.6% with *P*-value 0.000) (Fig. 5), no sources of heterogeneity were identified using subgroup Meta-analysis.

### Publication bias (Bias detection)

**Fig. 6:**
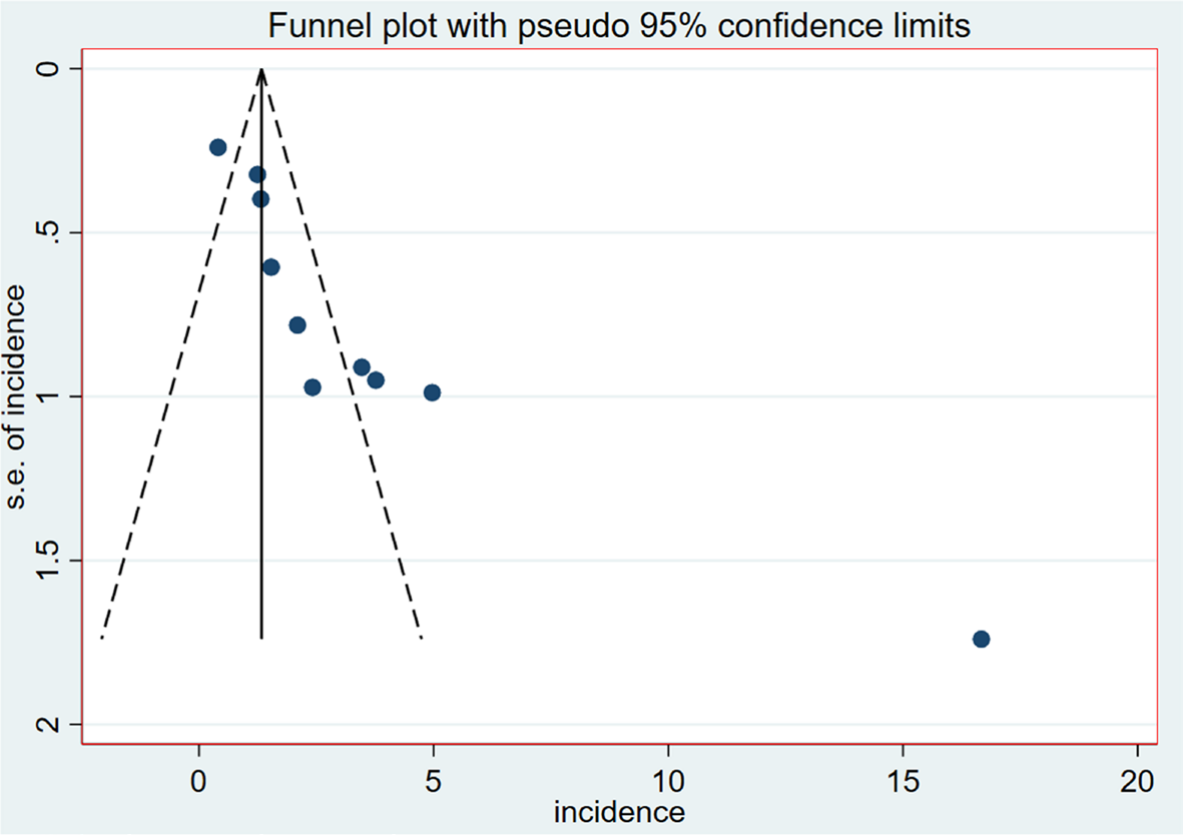
Publication bias check using funnel plot in the heterogeneity of first-line ART failure among Ethiopian children

The scatter plots were asymmetrical, indicating that the small-study had effects on the heterogeneity of first-line ART failure among Ethiopian children (Fig. 6). The Egger linear regression test was used to objectively assess the presence or absence of publication bias. As a result, there was statistically significant publication bias (*P* = 0.000). As a result, we conducted trim and fill analysis, demonstrating that publication was identified as the source of heterogeneity.

### Predictors of first-line ART failure

Baseline WHO stage (including five studies), adherence (including four studies), disclosure status (including three studies), baseline tuberculosis infection status (including two studies), follow-up duration (including four studies), and parental status were used to predict the onset of first-line ART failure in Ethiopian children (including three studies). First-line ART failure was associated with advanced WHO clinical stage at baseline, poor ART adherence, and having baseline TB-HIV coinfection, which were identified as significant predictors of first-line ART failure among children in Ethiopia.

**Fig. 7:**
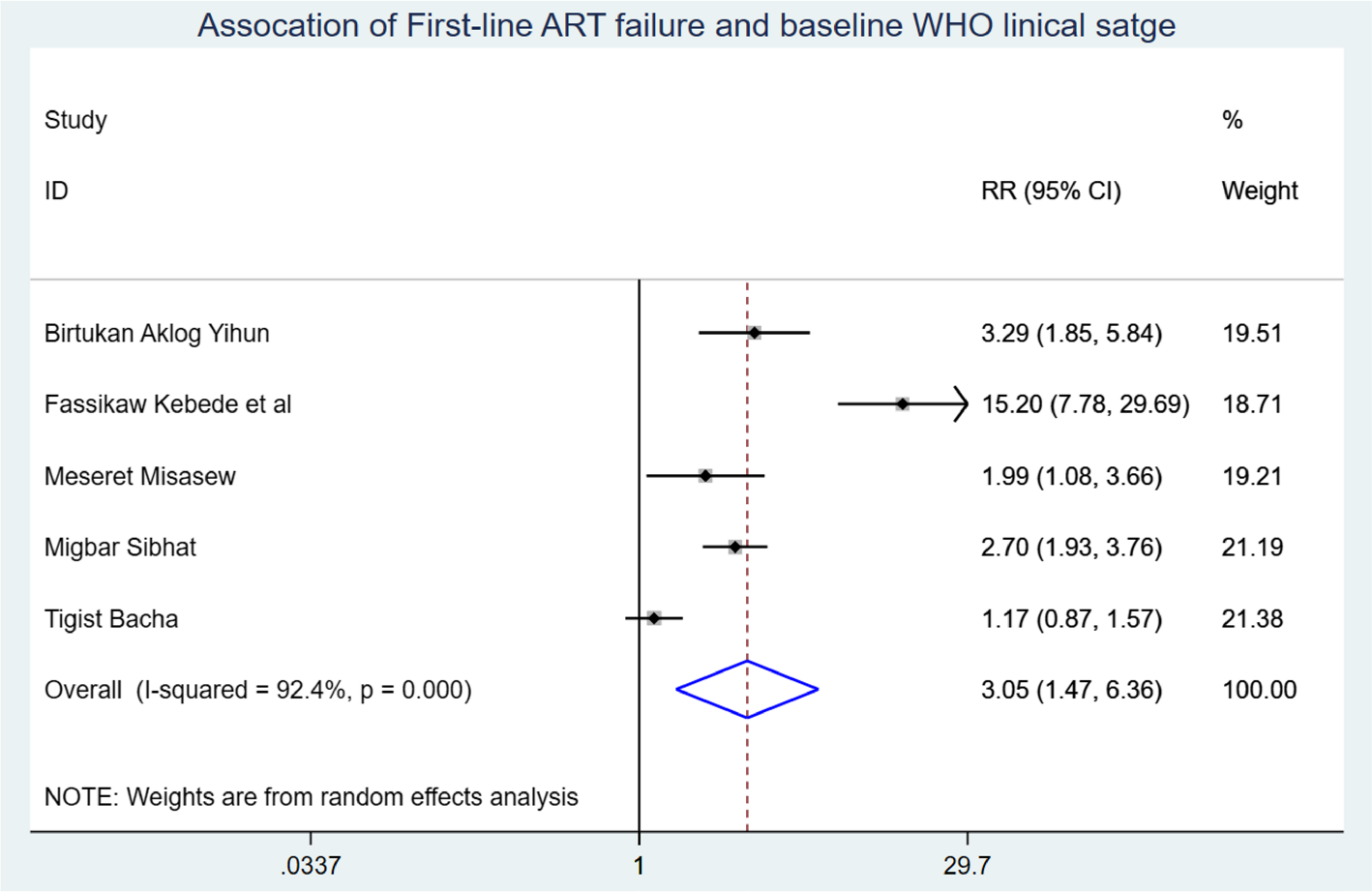
association of first-line ART failure and WHO clinical stage among Ethiopian children

**Fig. 8:**
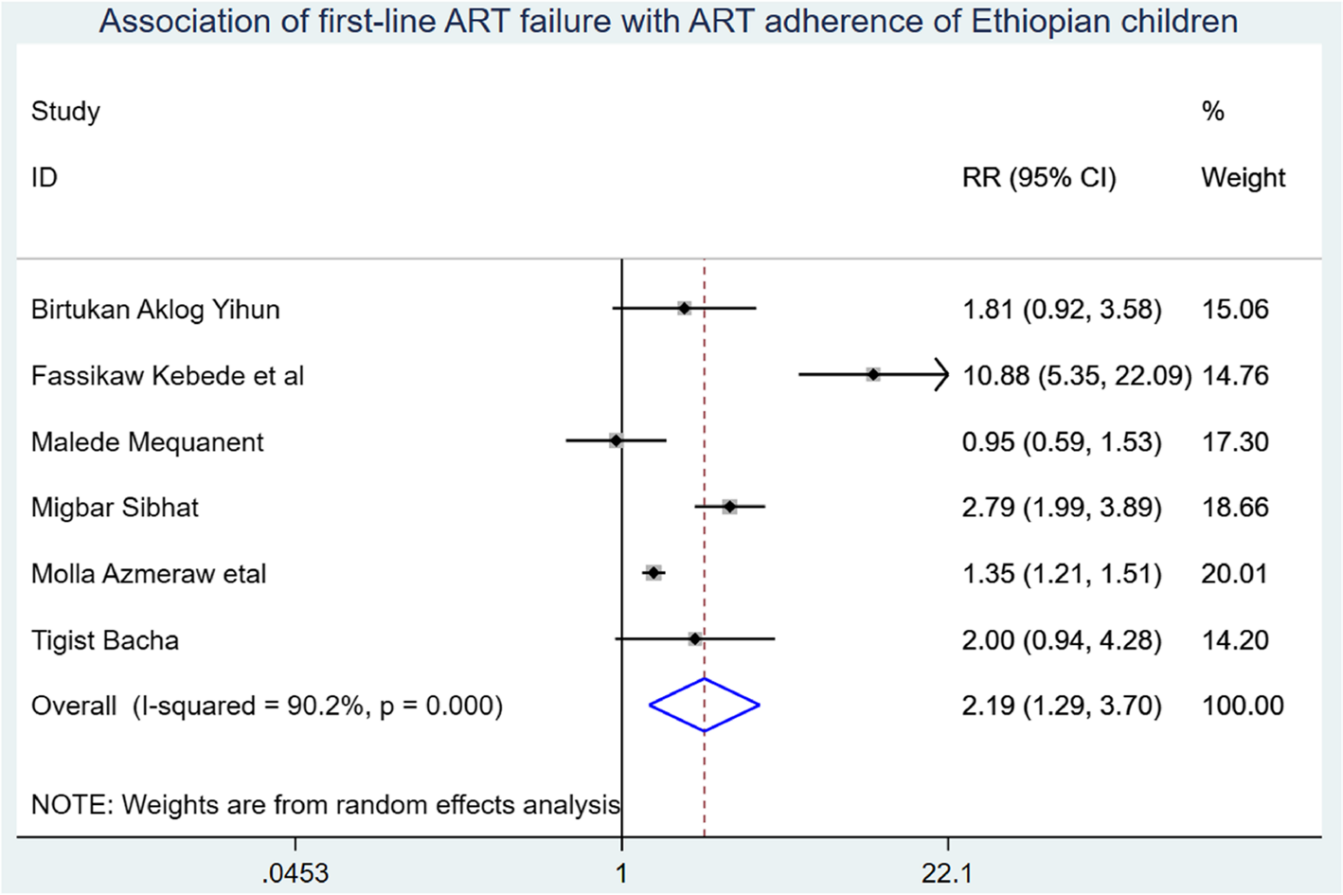
Association of first-line ART failure and ART adherence of Ethiopian Children

**Fig. 9:**
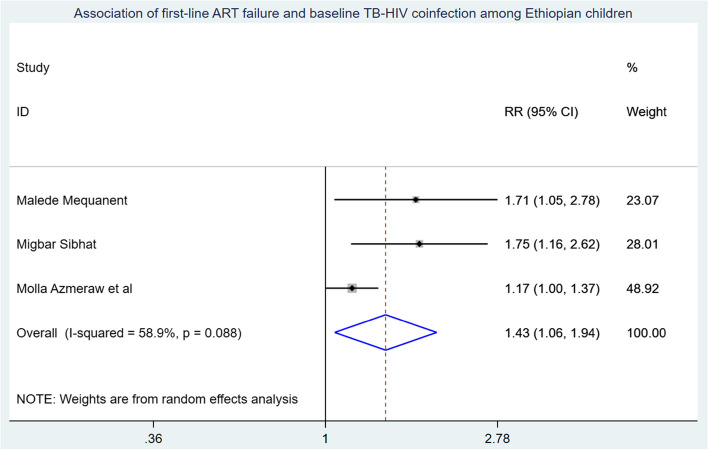
Association of first-line ART failure and TB-HIV coinfection among Ethiopian Children

Those study participants who began ART at an advanced WHO clinical stage had a 3.05 (95% CI: 1.47, 6.36) times higher chance of getting first-line ART failure when compared with those children who were on WHO clinical stage I & II (Fig. 7). Children who have poor ART adherence had a 2.79 (95% CI: 1.29, 3.70) times higher chance of experiencing first-line ART failure than children having good first-line ART adherence (Fig. 8). The likelihood of experiencing first-line ART failure among children having TB-HIV coinfection had 1.43 (95%CI: 1.06, 1.94) times higher than their counterparts (Fig. 9).

## Discussions

This systematic review and Meta-analysis included 10 articles having 5446 children in Ethiopia. The sample size of included studies ranged from 250 to 824 Ethiopian children.

The onset of first-line ART failure among Ethiopian children was 3.18 (95% CI: 1.91, 4.44) child-years of ART failure-free observations. It is high in order for meet the Joint UNAIDS treatment achievement of ending the HIV epidemic by 2030. This could be attributed to delayed ART initiation in HIV-infected children, insufficient focus on community HIV screening, and a lack of close follow-up for daily pill intake. These factors contribute to comorbidities, viral multiplication, low CD4 counts, and disease progression, ultimately leading to ART failure in Ethiopia. Children who were at an advanced WHO clinical stage when they began ART had a 3.05 times higher likelihood (95% CI: 1.47, 6.36) of experiencing first-line ART failure compared to their counterparts. It is supported by a study conducted in Cameroon and Tanzania [36, 37]. This could be attributed to delayed ART initiation in children, leading to a high viral load and low CD4 cell count, which in turn causes the deterioration of the child’s immune system and disease progression [38]. This ultimately results in first-line ART failure among Ethiopian school children on ART.

First-line ART failure experience among children who had poor ART adherence was 2.79 (95% CI: 1.29, 3.70) times higher than their counterparts. It is supported by a study conducted in Tanzania [37, 39]. This could be due to the fact that the benefits of ART are maximized when children have regular follow-ups and maintain good adherence. Poor adherence to ART is associated with a loss of sustained viral suppression, an increased risk of drug resistance, the emergence of resistant strains, and disease progression [40, 41].

Children with TB-HIV coinfection have a 1.92 times higher risk (95% CI: 1.06, 1.94) of first-line ART failure compared to those without TB-HIV coinfection. This increased risk may be due to overlapping drug toxicities, side effects, pill burden, drug-drug interactions, and the immune system weakening caused by TB. These factors can lead to the occurrence of IRIS, poor ART adherence, high viral replication, and disease progression, ultimately resulting in first-line ART failure [42].

### Conclusion and recommendation

The onset of first-line ART failure among Ethiopian children on ART was high to achieve ending AIDS epidemics by 2030. Advanced WHO stage, poor ART adherence, and TB-HIV coinfection at the initiation of ART were the identified predictors. Regarding ART initiation for children at an advanced WHO clinical stage, the Minister of Health should continue to strengthen community HIV screening and the test-and-treat program. Families and caregivers should collaborate in community HIV screening and ensure children are taken to an ART treatment center as soon as their HIV status is known. Addressing poor ART adherence: The Minister of Health should implement telemedicine initiatives, such as sending text message reminders for children to take their daily ART dose. Families should also remind and supervise their children during their daily ART intake. Regarding TB-HIV coinfection: The Minister of Health should ensure that TB investigation labs are well-equipped, and healthcare professionals should continue diagnosing TB in HIV-infected children during follow-ups. Non-governments organizations with collaborations with governmental organizations should offer a comprehensive package of interventions, including screening, treatment, and prophylaxis for major opportunistic infections, to reduce morbidity and mortality in patients with advanced HIV disease.

Healthcare professionals ensure that TB investigation labs are well-equipped and continue diagnosing TB in HIV-infected children during follow-ups.

The future researcher should conduct on early intervention studies focusing on the benefits of early ART initiation to prevent progression to advanced WHO clinical stages and Adherence Improvement Strategies to investigate effective strategies to improve ART adherence among children, such as community support programs.

## Supplementary Information

Additional file 1: PRISMA _2020_checklist_cohort.

## Data Availability

No datasets were generated or analysed during the current study.

## Abbreviations

AIDS: Acquired immunodeficiency syndrome
HIV: Human Immunodeficiency Virus
UNAIDS: United Nations Program on HIV/AIDS
CLHIV: Children Living with Human Immunodeficiency Virus
ART: Antiretroviral Therapy
AZT: Zidovudine
ABC: Abacavir
3TC: Lamivudine
EFV: Efavirenz
NVP: Nelfinavir
TDF: Tenofovir
